# Size dependent optical temperature sensing properties of Y_2_O_3_: Tb^3+^, Eu^3+^ nanophosphors

**DOI:** 10.1039/c8ra10066g

**Published:** 2019-01-18

**Authors:** Lixin Peng, Qingyu Meng, Wenjun Sun

**Affiliations:** Key Laboratory for Photonic and Electronic Bandgap Materials, Ministry of Education, School of Physics and Electronic Engineering, Harbin Normal University Harbin 150025 China qingyumeng163@163.com

## Abstract

Using urea as a precipitation agent, Tb^3+^, Eu^3+^ co-doped Y_2_O_3_ nanophosphors were synthesized by a homogeneous precipitation method. The sizes of the sample particles were controlled by changing the molar ratio of the urea and rare earth ions. The microstructure and crystallographic structure of the sample were determined through powder X-ray diffraction (PXRD) and field emission scanning electron microscopy (FE-SEM). The test results show that the sample is body centered cubic. As the molar ratio of urea to rare earth ions increases, the size of the sample particles decreases. The temperature-dependent emission spectra of Tb^3+^, Eu^3+^ co-doped Y_2_O_3_ phosphors with different particle sizes were measured. The results showed that because the fluorescence intensity ratio (FIR) of Tb^3+^ and Eu^3+^ varies with temperature, it can be used to visually reflect changes in temperature. In addition, the temperature sensing sensitivity of Tb^3+^ and Eu^3+^ co-doped Y_2_O_3_ phosphors increased upon a decrease in the particle size, but the relative sensitivity decreased with a decrease in the particle size. The physical mechanism of the sensitivity and relative sensitivity changes with the size of the sample particles was also explained.

## Abstract 2

Using urea as a precipitation agent, Tb^3+^, Eu^3+^ co-doped Y_2_O_3_ nanophosphors were synthesized by a homogeneous precipitation method. The size dependence-optical temperature sensing properties of nanophosphors have been studied.

## Graphical abstract

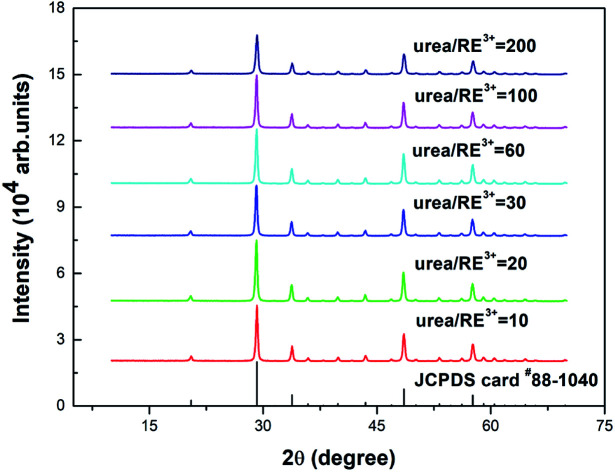

## 1. Introduction

In terms of fluorescent rare earth ions, optical temperature measurement has received widespread attention in recent years.^1–6^ The reason for this is that by studying the relationship between fluorescence intensity and ambient temperature, non-contact temperature measurement can be achieved. Compared with common temperature detection equipment, the optical temperature sensor based on fluorescent rare earth ions does not change the original temperature of the measured object. Fluorescence intensity ratio (FIR) technology is a high precision optical temperature measurement method, using the luminescence intensity ratio of two rare earth ions to determine the temperature. FIR is not affected by spectrum losses and excitation source fluctuation, so it can be widely used.^7–10^ As is known, Er^3+^-activated FIR temperature sensing materials are the most common of the rare earth-containing temperature sensors. However, when the up-conversion luminescence of Er^3+^ is excited by a NIR-laser, a strong thermal effect occurs that affects the accuracy of any measurement. In addition, the luminescence colors of the two thermal energy levels of Er^3+^ are both green, therefore the luminescence color cannot visually reflect any changes in temperature.^11–13^ However, the ^5^D_4_–^7^F_5_ transition of Tb^3+^ produces green luminescence, and the ^5^D_0_–^7^F_2_ transition of Eu^3+^ produces red luminescence.^14,15^ If the thermal-quenching trends of luminescence of Tb^3+^ and Eu^3+^ are different in the same host, the FIR of Tb^3+^ and Eu^3+^ will change with temperature and therefore, the temperature change can be directly reflected by the change in the luminescence color. In addition, a Eu^3+^ and Tb^3+^ co-activated material is excited by fluorescence, without the occurrence of any thermal effect. Thus, it is promising to study Tb^3+^ and Eu^3+^ co-doped materials.^16–18^

The selection of suitable host materials is very important for the preparation of an optical temperature measurement material. Y_2_O_3_ is a host material that has been studied widely in recent years. Y_2_O_3_ has optical inertia, good biocompatibility, low phonon energy, physical and chemical stability and thermal stability. Moreover, it can be easily doped with rare earth metals and is an ideal host material.^19,20^ For the above reasons, a series of Y_2_O_3_: Tb^3+^, Eu^3+^ phosphors were synthesized using a homogeneous precipitation method. By changing the molar ratio of urea and rare earth ions, Tb^3+^, Eu^3+^ co-doped Y_2_O_3_ phosphors with different particle sizes were prepared. The dependence between the optical temperature sensing characteristics of the samples and particle size was determined. The physical mechanism of sensitivity and relative sensitivity changes with the size of sample particles is also explained.

## 2. Experimental

### 2.1 Synthesis

Using urea (CO(NH_2_)_2_) as a precipitation agent, a series of Y_2_O_3_: Tb^3+^, Eu^3+^ phosphors were synthesized by a homogeneous precipitation method. The reaction conditions were optimized by referring to the experimental parameters of previously synthesized materials in the literature.^21–24^ Firstly, 0.05 mmol of Eu(NO_3_)_3_, 9.45 mmol of Y(NO_3_)_3_ and 0.5 mmol of Tb(NO_3_)_3_ were put into a 250 ml beaker, and 120 ml of deionized water was added to form a clear aqueous solution under stirring. Then, 40 ml of an aqueous solution of urea (the urea : RE^3+^ ratios in the different experiments were 10, 20, 30, 60, 100 and 200, respectively) was slowly added to the above solution, which was then stirred for 20 min to form a clear aqueous solution. Next, the temperature of the oven was raised to 90 °C in advance, and beaker with the solution in was placed in the oven and heated for 5 h, after which the beaker was removed and allowed to cool to room temperature. White precipitate products were observed in the beaker in the different experiments. The precipitated products were centrifuged and washed several times, then dried at 80 °C for 5 h. Finally, the precipitated products were calcined at a high temperature of 850 °C for 2 h under a nitrogen atmosphere to obtain the Y_2_O_3_: 5% Tb^3+^, 0.5% Eu^3+^ phosphors.

### 2.2 Characterization

Powder X-ray diffraction (PXRD) patterns of the samples were obtained on a Rigaku D/max2600 (*λ* = 0.15406 nm) diffractometer equipped with a Cu Kα1 radiation source. The data were collected in the range of 10–70° with a scanning step of 0.02° and a scanning rate of 4.0° min^−1^. The size, shape and structure of the samples were characterized by emission scanning electron microscopy (Hitachi SU70 FE-SEM). The emission spectra, excitation spectra and fluorescence decay curves were measured using an Edinburgh FLS-920 fluorescence spectrometer, with a 450 W Xenon lamp as the excitation light source. Temperature control was achieved by an Orient KOJI TAP-02 high temperature fluorescence controller (including a FLS-920 matched sample chamber and sample holder), over a temperature range of 313 to 513 K, with a temperature control accuracy of 0.1 °C. In the experiments, the sample was kept at temperature for 2 min after the temperature control device reached the target temperature, then the measurements were started with the aim of ensuring that the sample temperature was the same as the target temperature.

## 3. Results and discussion

### 3.1 The microstructures and crystal structures of the phosphors

**Fig. 1:**
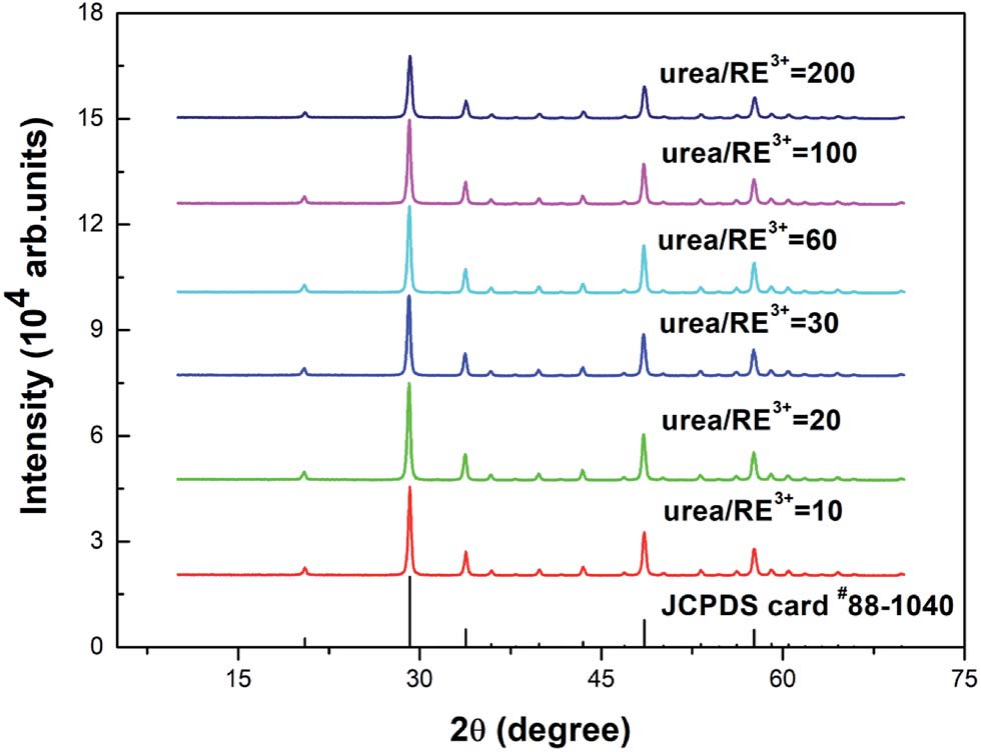
PXRD patterns of 5% Tb^3+^, 0.5% Eu^3+^ co-doped Y_2_O_3_ phosphors with different urea and RE^3+^ molar ratios of 10, 20, 30, 60, 100 and 200.

Fig. 1 shows the powder X-ray diffraction (PXRD) spectra of the Y_2_O_3_: Tb^3+^, Eu^3+^ phosphors prepared using different molar ratios of urea and rare earth ions of 10, 20, 30, 60, 100 and 200. As shown in Fig. 1, the diffraction peaks of the prepared samples are all consistent with the JCPDS card #88-1040 and the crystal lattice is body centered cubic.

**Fig. 2:**
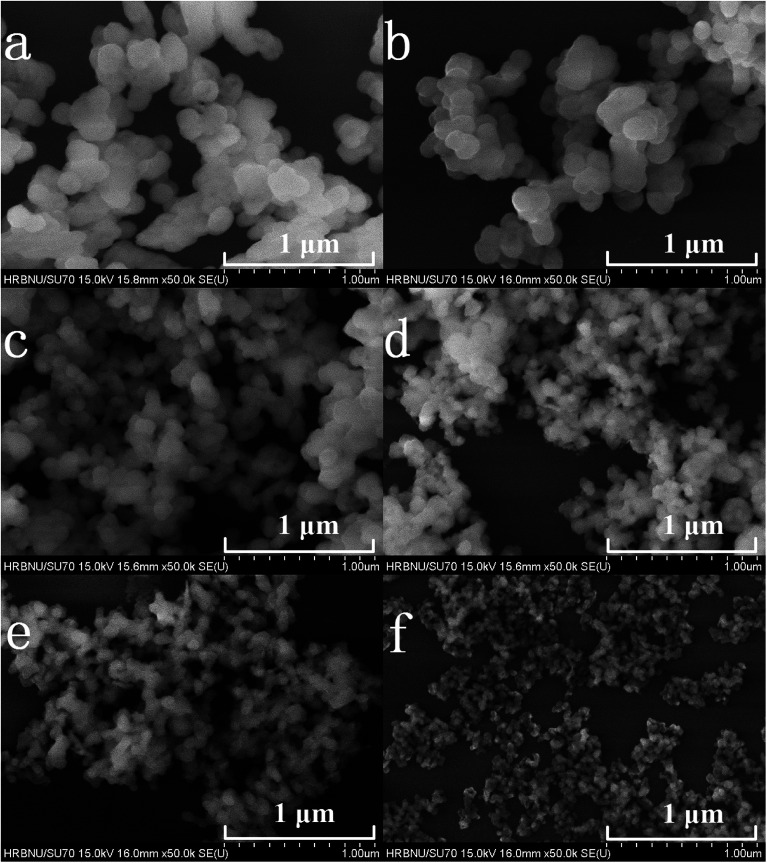
FE-SEM images of 5% Tb^3+^, 0.5% Eu^3+^ co-doped Y_2_O_3_ phosphors with different urea and RE^3+^ molar ratios of (a) 10, (b) 20, (c) 30, (d) 60, (e) 100 and (f) 200.

Fig. 2(a–f) shows the FE-SEM images of the prepared samples. It can be clearly observed from Fig. 2 that the particle sizes of the Y_2_O_3_ phosphors decrease upon an increase in the molar ratio of urea to rare earth ions (the molar ratios of urea and rare earth ions are (a) 10, (b) 20, (c) 30, (d) 60, (e) 100 and (f) 200, respectively).

**Fig. 3:**
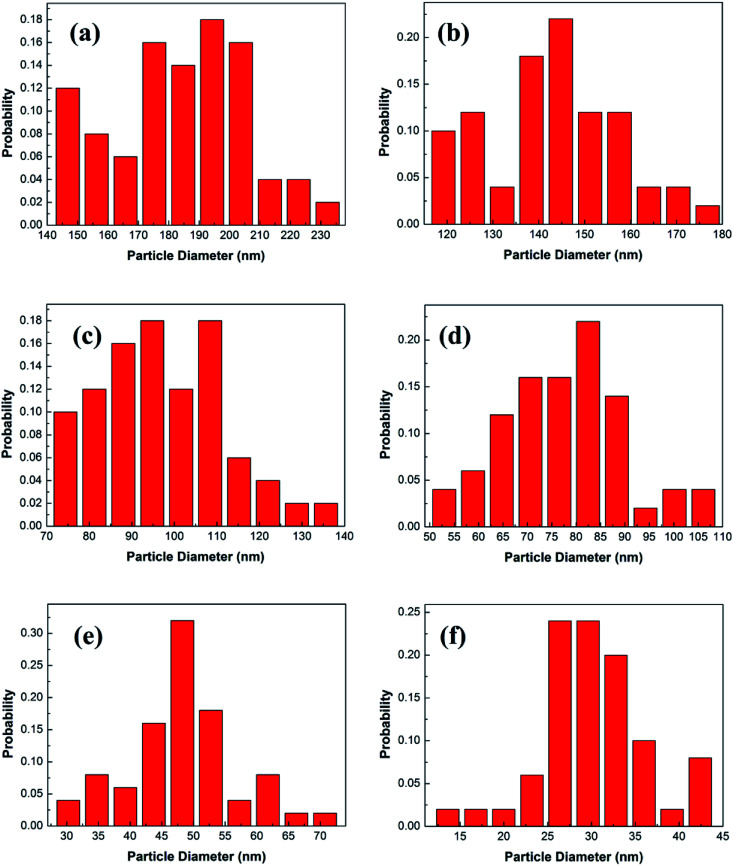
Particle diameter distributions of 5% Tb^3+^, 0.5% Eu^3+^ co-doped Y_2_O_3_ phosphors with different urea and RE^3+^ molar ratios of (a) 10, (b) 20, (c) 30, (d) 60, (e) 100 and (f) 200.

Fig. 3 shows the statistical results of the particle sizes. The average particle size is calculated by the statistical particle size data. The average particle diameters of the Y_2_O_3_: Tb^3+^, Eu^3+^ phosphors are 184.02, 142.88, 97.37, 78.25, 48.46 and 30.27 nm, for molar ratios of urea and rare earth ions of 10, 20, 30, 60, 100 and 200, respectively. Therefore, the particle sizes were estimated to be about 200, 150, 100, 80, 50 and 30 nm, respectively.

### 3.2 Temperature sensing characteristics of the Y_2_O_3_: 5% Tb^3+^, 0.5% Eu^3+^ phosphors

**Fig. 4:**
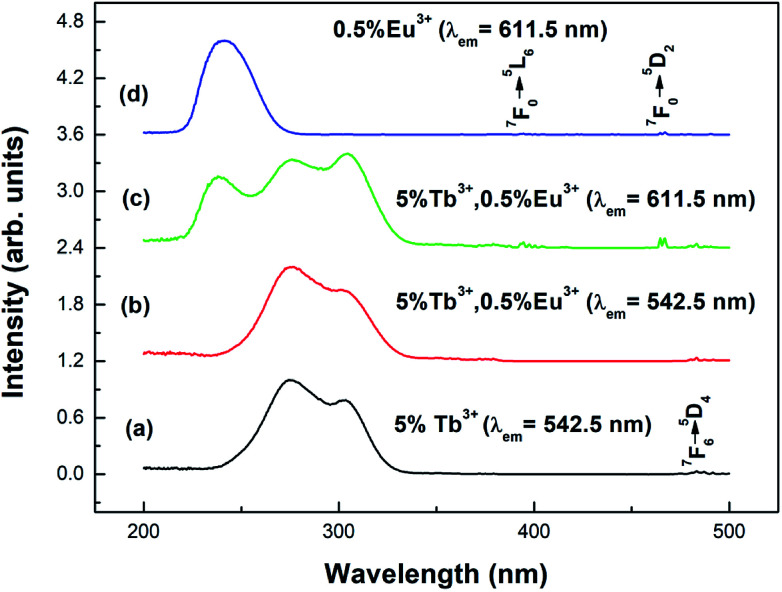
Excitation spectra of different samples with a particle size of 100 nm: (a) Y_2_O_3_: 5% Tb^3+^ (*λ*_em_ = 542.5 nm); (b) Y_2_O_3_: 5% Tb^3+^, 0.5% Eu^3+^ (*λ*_em_ = 542.5 nm); (c) Y_2_O_3_: 5% Tb^3+^, 0.5% Eu^3+^ (*λ*_em_ = 611.5 nm); (d) Y_2_O_3_: 0.5% Eu^3+^ (*λ*_em_ = 611.5 nm).

Fig. 4 shows the excitation spectra of the prepared samples (the particle size is 100 nm) at room temperature. Curve (a) shows the excitation spectrum of the Y_2_O_3_: 5% Tb^3+^ phosphor, at a monitoring wavelength of 542.5 nm (consistent with the Tb^3+ 5^D_4_ → ^7^F_5_ transition). From curve (a), it can be seen that there is a strong excitation band in the range of 230–340 nm, which corresponds to the absorption transition of Tb^3+^ 4f–5d.^25^ At 484 nm, there is a weak excitation peak corresponding to Tb^3+ 7^F_6_–^5^D_4_.^26^ Curve (d) shows the excitation spectrum of the Y_2_O_3_: 0.5% Eu^3+^ phosphor, at a monitoring wavelength of 611.5 nm (consistent with the Eu^3+ 5^D_0_ → ^7^F_2_ transition). From curve (d), it can be seen that there is a strong excitation band at 210–280 nm corresponding to the charge transition absorption band of O^2−^–Eu^3+^.^27^ At 394.5 and 465 nm, there are two weak excitation peaks corresponding to the ^7^F_0_ → ^5^L_6_ and ^7^F_0_ → ^5^D_2_ transitions of Eu^3+^.^26^

Curves (b) and (c) show the excitation spectra of Y_2_O_3_: 5% Tb^3+^, 0.5% Eu^3+^ monitored at 542.5 nm and 611.5 nm. As can be seen from curve (b), when the luminescence of Tb^3+^ was monitored at 542.5 nm, no characteristic excitation was observed for Eu^3+^. However, as seen in curve (c), when monitoring the luminescence of Eu^3+^ at 611.5 nm, it was found that the Tb^3+^ (4f–5d) characteristic excitation band was particularly obvious. This indicates that the energy transfer from Eu^3+^ to Tb^3+^ is invalid, but the energy transfer from Tb^3+^ to Eu^3+^ is effective.^28^

**Fig. 5:**
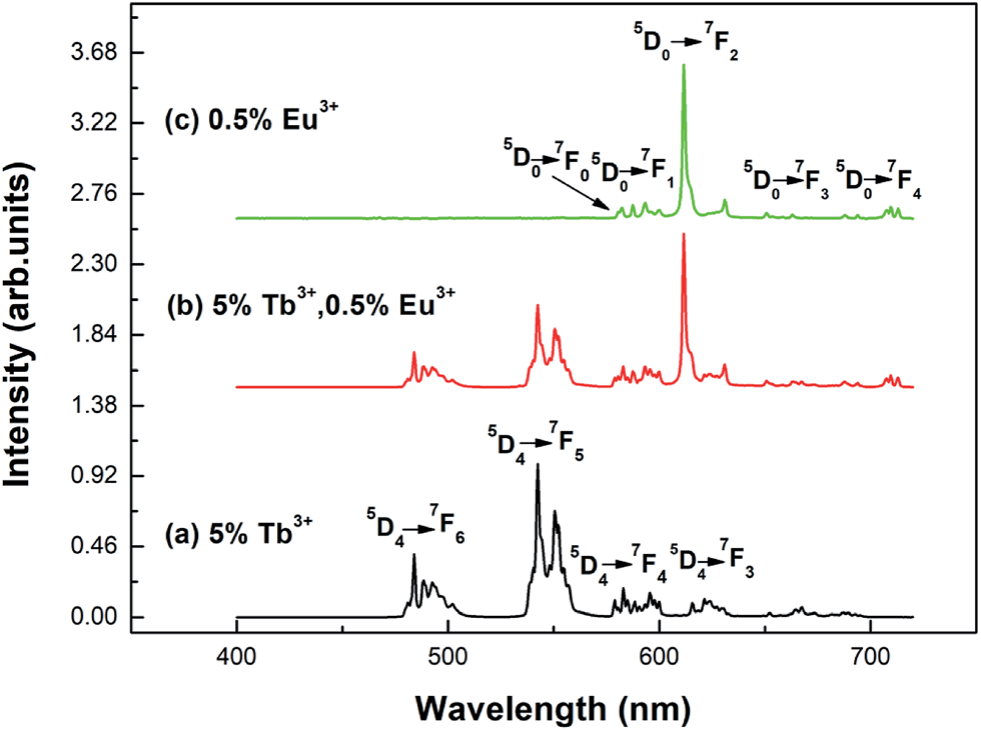
Emission spectra of different samples with a particle size of 100 nm: (a) Y_2_O_3_: 5% Tb^3+^ (*λ*_ex_ = 276 nm); (b) Y_2_O_3_: 5% Tb^3+^, 0.5% Eu^3+^ (*λ*_ex_ = 276 nm); (c) Y_2_O_3_: 0.5% Eu^3+^ (*λ*_ex_ = 240 nm).

Fig. 5 shows the emission spectra of the different samples at room temperature. Curves (a) and (b) show the emission spectra of the Y_2_O_3_: 5% Tb^3+^ and Y_2_O_3_: 5% Tb^3+^, 0.5% Eu^3+^ phosphors, at an excitation wavelength of 276 nm. Curve (c) shows the emission spectrum of the Y_2_O_3_: 0.5% Eu^3+^ phosphor at an excitation wavelength of 240 nm. The particle sizes of all three samples were 100 nm. Curve (a) shows the Tb^3+^ 4f–4f transition lines located at 484 nm (^5^D_4_ → ^7^F_6_), 542.5 nm (^5^D_4_ → ^7^F_5_), 583 nm (^5^D_4_ → ^7^F_4_), and 621 nm (^5^D_4_ → ^7^F_3_).^25^ Curve (c) shows the Eu^3+^ 4f–4f transitions, 580 nm (^5^D_0_ → ^7^F_0_), 593 nm (^5^D_0_ → ^7^F_1_), 611.5 nm (^5^D_0_ → ^7^F_2_), 651 nm (^5^D_0_ → ^7^F_3_), and 710 nm (^5^D_0_ → ^7^F_4_).^29^ Curve (b) shows the Eu^3+^ 4f–4f transition and Tb^3+^ 4f–4f transition emission peaks.

**Fig. 6:**
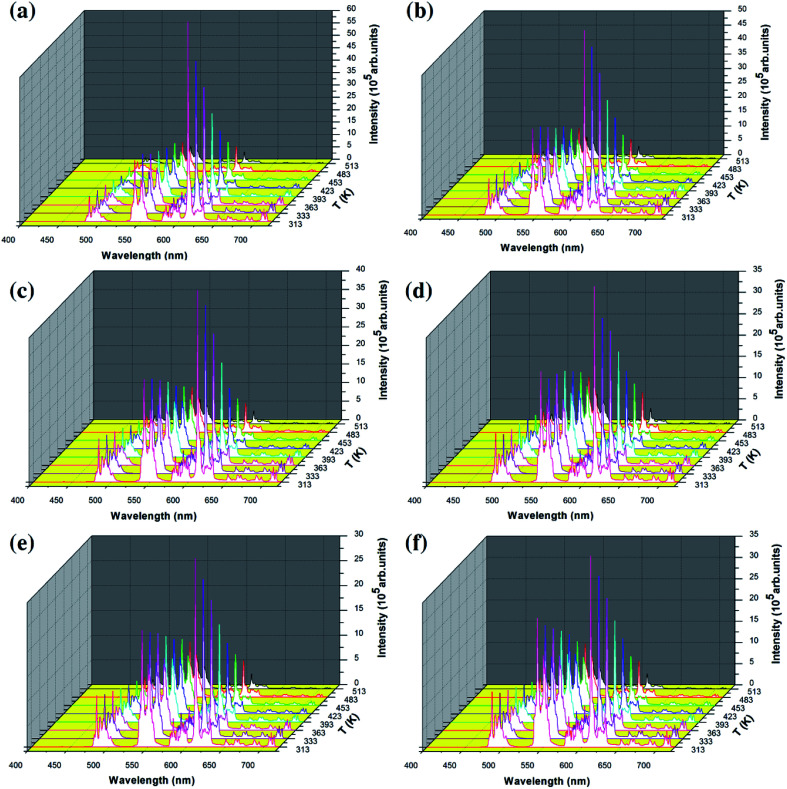
Temperature-dependent emission spectra of all of the samples by 276 nm excitation: (a–f) Y_2_O_3_: 5% Tb^3+^, 0.5% Eu^3+^ (the particle sizes of the samples were (a) 200 nm, (b) 150 nm, (c) 100 nm, (d) 80 nm, (e) 50 nm and (f) 30 nm, respectively).

In order to study the temperature sensing properties of the material in more depth, the temperature-dependent emission spectra of Y_2_O_3_: 5% Tb^3+^, 0.5% Eu^3+^ (the particle sizes of the samples are 200 nm, 150 nm, 100 nm, 80 nm, 50 nm and 30 nm, respectively) were measured in the temperature range of 313–513 K. Fig. 6(a–f) shows the temperature-dependent emission spectra of the samples at an excitation wavelength of 276 nm. In Fig. 6(a–f), it can be seen that the luminescence intensity of Tb^3+^ and Eu^3+^ decrease gradually as the temperature increases. However, the luminescence intensity of Eu^3+^ decreased more significantly. So, the FIR of Tb^3+^ to Eu^3+^ was observed to increase upon an increase in the temperature and therefore, the temperature change can be directly reflected by the FIR of Tb^3+^ and Eu^3+^.

For a single doped material, the relationship between the luminescence intensity and temperature can be expressed by eqn (1):^30,31^1
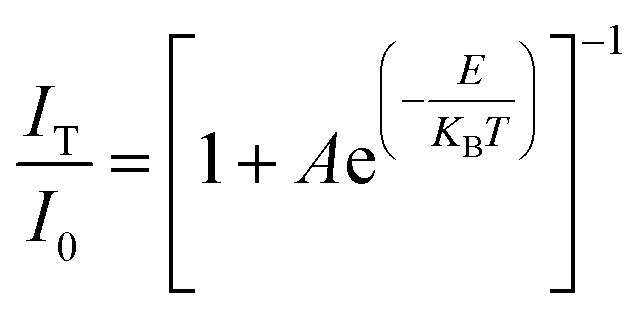


In eqn (1), *I*_0_ is the luminescence intensity at 0 K, *I*_T_ is the luminescence intensity at a specific temperature *T*, *A* is a constant, *E* is the heat quenching activation energy, *K*_B_ is the Boltzmann constant and *T* is the absolute temperature.

In the co-doped Tb^3+^ and Eu^3+^ samples, the FIR of Tb^3+^ (^5^D_4_–^7^F_5_) to Eu^3+^ (^5^D_0_–^7^F_2_) in the variable temperature emission spectrum can be expressed as eqn (2), where the value of FIR be defined as *R*.2
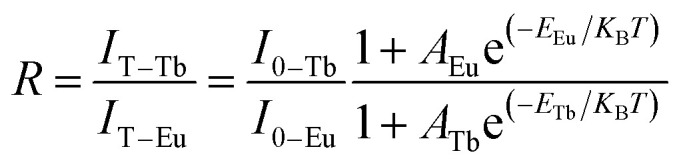


By simplifying the derivation of eqn (2), a new formula *R* emerges:^32^

**Fig. 7:**
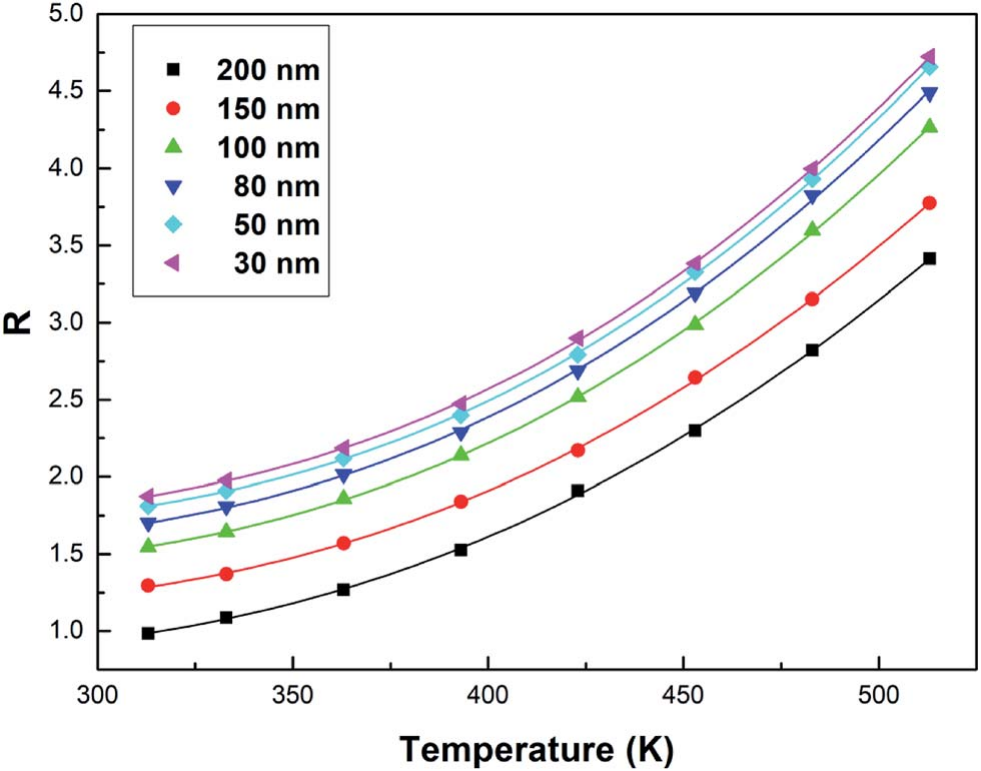
Curve *R versus* temperature in the Tb^3+^, Eu^3+^ co-doped Y_2_O_3_ phosphors (the particle sizes of the samples were 200 nm, 150 nm, 100 nm, 80 nm, 50 nm and 30 nm, respectively), the solid points represent the experimental data and the solid lines show the fitting result using eqn (3).

3 *R* ≈ *B* + *C*e^−Δ*E*/*K*_B_*T*^

where *B*, *C*, and Δ*E* are parameters related to the *I*_0_, *A*, and *E* values of Tb^3+^ and Eu^3+^. The values of *B*, *C* and Δ*E*/*K*_B_ are acquired *via* the fitting of the experimental data to eqn (3), the results of which are shown in Fig. 7. In this figure, the points represent the experimental data, and the lines show the fitting data. The *B*, *C* and Δ*E*/*K*_B_ values of the samples with different particle sizes are listed in Table 1.

**Table 1:** *B*, *C*, and Δ*E*/*K* values of different particle size samples, and their correlation coefficients, *R*^2^

Particle size	*B*	*C*	Δ*E*/*K*_B_ (K)	*R* ^2^
200 nm	0.800 ± 0.021	163.305 ± 14.164	2121.319 ± 47.073	0.99979
150 nm	1.112 ± 0.016	189.417 ± 13.023	2187.751 ± 37.164	0.99987
100 nm	1.369 ± 0.012	223.175 ± 11.418	2227.907 ± 27.593	0.99993
80 nm	1.521 ± 0.023	237.407 ± 21.680	2245.071 ± 49.209	0.99978
50 nm	1.633 ± 0.018	247.951 ± 17.875	2260.916 ± 38.814	0.99986
30 nm	1.692 ± 0.022	250.347 ± 22.385	2264.333 ± 48.134	0.99979

From Fig. 7, the FIR can be seen to increase as the particle size of the sample decreases at the same temperature. This is due to size limitation effects (also known as the volume effect) of the interfaces of nanomaterials, which inhibits the energy transfer from Tb^3+^ to Eu^3+^ in the samples.^33^ Upon a decrease in the particle size of the sample, the inhibition becomes more obvious in terms of energy transfer. So, the luminescence intensity of Tb^3+^ relatively increases and the luminescence intensity of Eu^3+^ relatively decreases in the emission spectra, and the FIR of Tb^3+^ to Eu^3+^ increases.

**Fig. 8:**
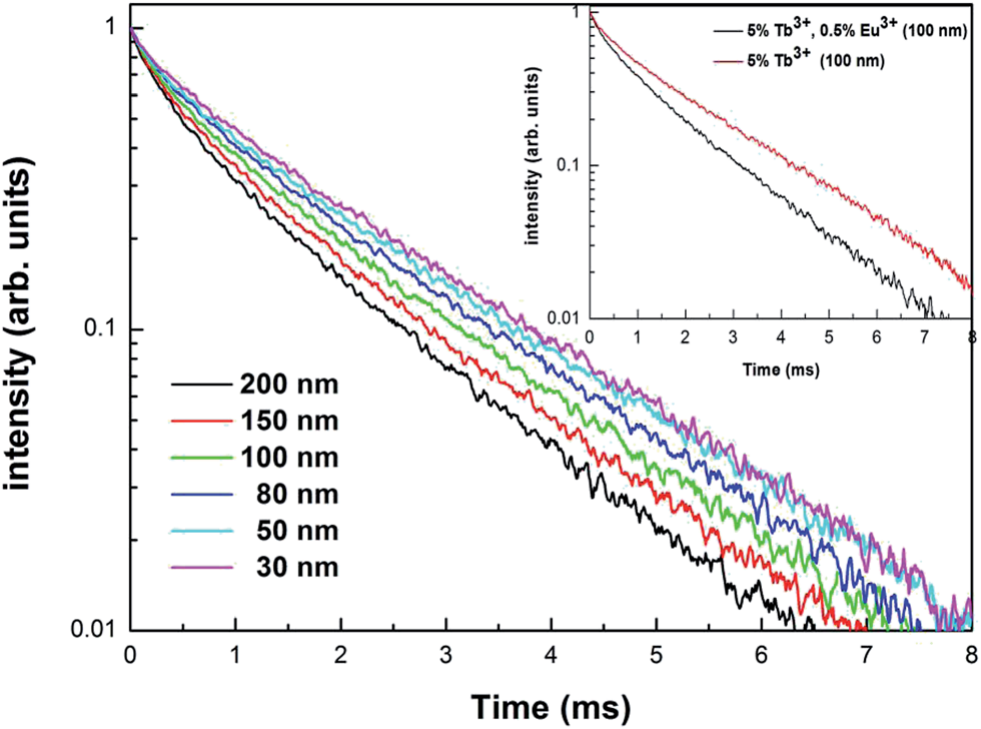
Fluorescence decay curves of the Tb^3+^ in Y_2_O_3_: 5% Tb^3+^, 0.5% Eu^3+^ phosphors (the particle sizes of the samples were 200 nm, 150 nm, 100 nm, 80 nm, 50 nm and 30 nm, respectively) monitoring 542.5 nm, by 276 nm excitation. The image shows the fluorescence decay curves of Tb^3+^ in Y_2_O_3_: 5% Tb^3+^ and Y_2_O_3_: 5% Tb^3+^, 0.5% Eu^3+^ phosphors with a particle size of 100 nm.

In order to further demonstrate the impact of the size limitation effects on the energy transfer, as shown in Fig. 8, the fluorescence decay curves of Tb^3+^ at ^5^D_4_–^7^F_5_ (542.5 nm) in different samples was measured at an excitation of 276 nm. As can be seen from Fig. 8, due to the existence of energy transfer from Tb^3+^ to Eu^3+^, the luminescence decay curve of Tb^3+^ no longer features a simple single exponential change, but instead exhibits a non-exponential form. So, the average fluorescence lifetime can be used to indicate the fluorescence decay. Therefore, the average fluorescence lifetime *τ*_m_ can be defined as:^34^4
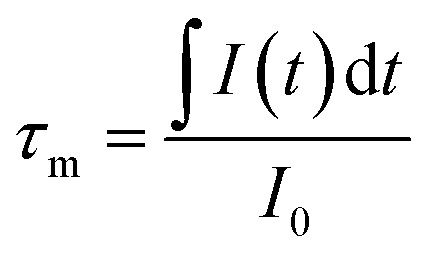
where, *I*_0_ is the luminescence intensity at the initial moment, and *I*(*t*) is the luminescence intensity at moment *t*. Using eqn (4), it was found that the fluorescence lifetimes of Tb^3+^ in the samples were 0.988 ms (200 nm), 1.096 ms (150 nm), 1.206 ms (100 nm), 1.321 ms (80 nm), 1.408 ms (50 nm) and 1.489 ms (30 nm), respectively. From these data, it can be clearly seen that the fluorescence lifetime of Tb^3+^ becomes longer as the particle size of the sample decreases, which is due to the influence of size limitation effects on the energy transfer, meaning that the energy transfer of Tb^3+^ to Eu^3+^ was inhibited, and the nonradiative relaxation rate of Tb^3+^ was reduced, therefore the fluorescence lifetime of Tb^3+^ is longer for a smaller sized sample than for a larger sized sample.

Fig. 8 shows the fluorescence decay curves of Tb^3+^ in the Y_2_O_3_: 5% Tb^3+^, 0.5% Eu^3+^ and Y_2_O_3_: 5% Tb^3+^ phosphors, where the particle size of the samples was 100 nm. The fluorescence lifetimes of Tb^3+^ in the co-doped and single-doped phosphors was 1.206 and 1.611 ms, respectively. It can be seen that the fluorescence lifetime of Tb^3+^ in single-doped phosphors is significantly longer than that of co-doped phosphors, which further indicates that there is energy transfer from Tb^3+^ to Eu^3+^.^35^

For practical application, sensitivity is widely used to evaluate the temperature measurement performance of optical temperature sensing. The greater the sensitivity, the more suitable a material is to be used as a temperature sensing material. Sensitivity is defined as the rate of change of FIR with temperature, which can be expressed as:^32^5
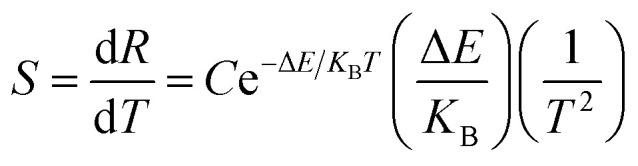


**Fig. 9:**
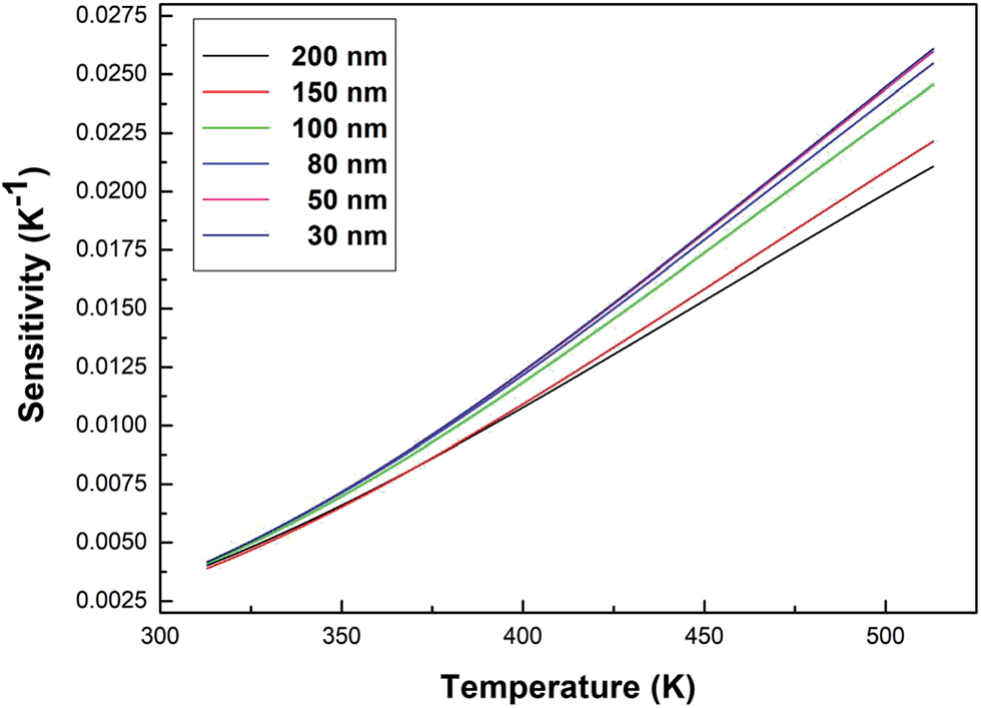
Curves of sensitivity *versus* temperature for the Tb^3+^, Eu^3+^ co-doped Y_2_O_3_ phosphors (the particle sizes of the samples were 200 nm, 150 nm, 100 nm, 80 nm, 50 nm and 30 nm, respectively).

Fig. 9 shows the sensitivity (*S*) of samples with different particle sizes over a temperature range of 313–513 K. As can be clearly seen from Fig. 9, the sensitivity maxima were 0.0212 K^−1^ (200 nm), 0.0222 K^−1^ (150 nm), 0.0246 K^−1^ (100 nm), 0.0255 K^−1^ (80 nm), 0.0260 K^−1^ (50 nm) and 0.0261 K^−1^ (30 nm) at 513 K, respectively. When the temperature is the same, the smaller the particle size of the sample, the greater the sensitivity of the sample. When the particle size of the sample decreases, the surface defects in the sample increase and the phonon vibration mode increases, increasing the coupling strength between electrons and phonons, which makes the luminescence properties of the sample more sensitive to temperature, therefore increasing the sensitivity of the sample. It can be concluded that the sensitivity can be improved by reducing the particle sizes of the samples.

The relative sensitivity can be represented by the following formula:^32^6
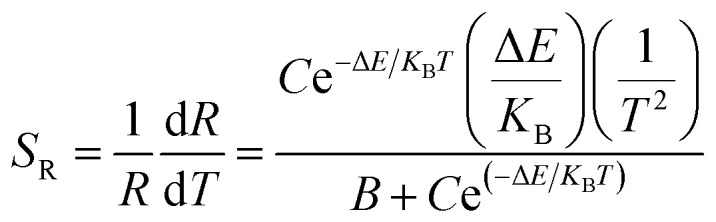


**Fig. 10:**
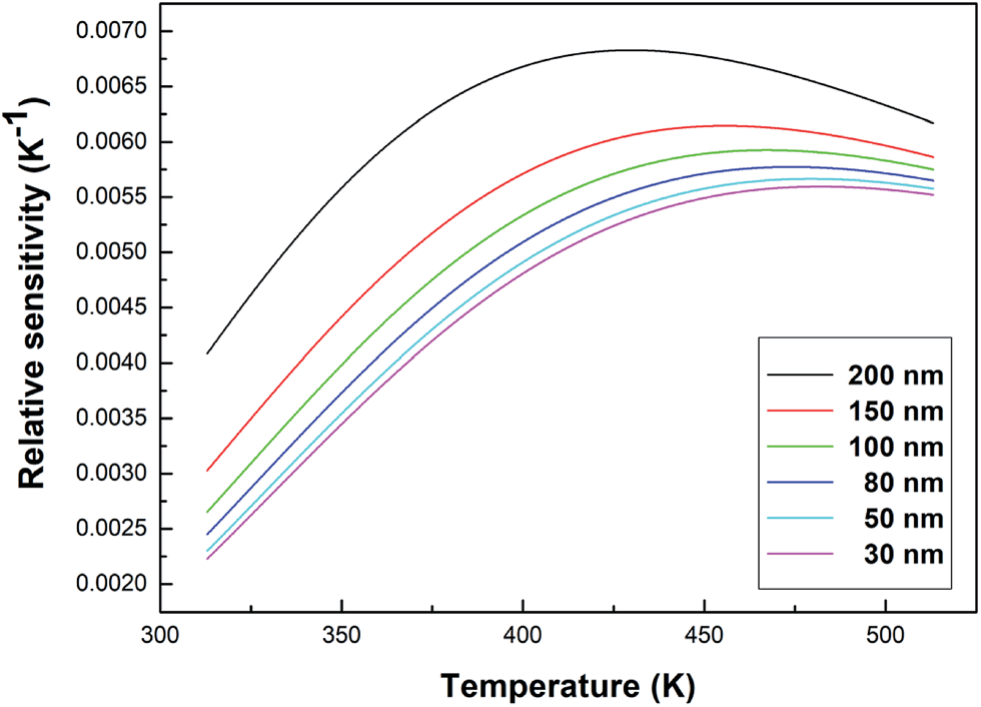
Curves of relative sensitivity *versus* temperature for the Tb^3+^, Eu^3+^ co-doped Y_2_O_3_ phosphors (the particle sizes of the samples were 200 nm, 150 nm, 100 nm, 80 nm, 50 nm and 30 nm, respectively).

As can be seen from Fig. 10, the relative sensitivity image of this sample presents a nonmonotonic trend. As the temperature increases, the relative sensitivity of each sample first increases and then decreases. As can be clearly observed in Fig. 10, at the same temperature, the relative sensitivity decreases as the particle size decreases. The relative sensitivity maxima were 0.00683 K^−1^ (200 nm, 430 K), 0.00615 K^−1^ (150 nm, 456 K), 0.00593 K^−1^ (100 nm, 469 K), 0.00577 K^−1^ (80 nm, 475 K), 0.00567 K^−1^ (50 nm, 479 K) and 0.00560 K^−1^ (30 nm, 483 K), respectively, due to the obvious increase in the *R* value with a decrease in the particle size. And, the value of *S* increased slightly, so according to eqn (6), the *S*_R_ value decreases upon a decrease in the particle size.

**Fig. 11:**
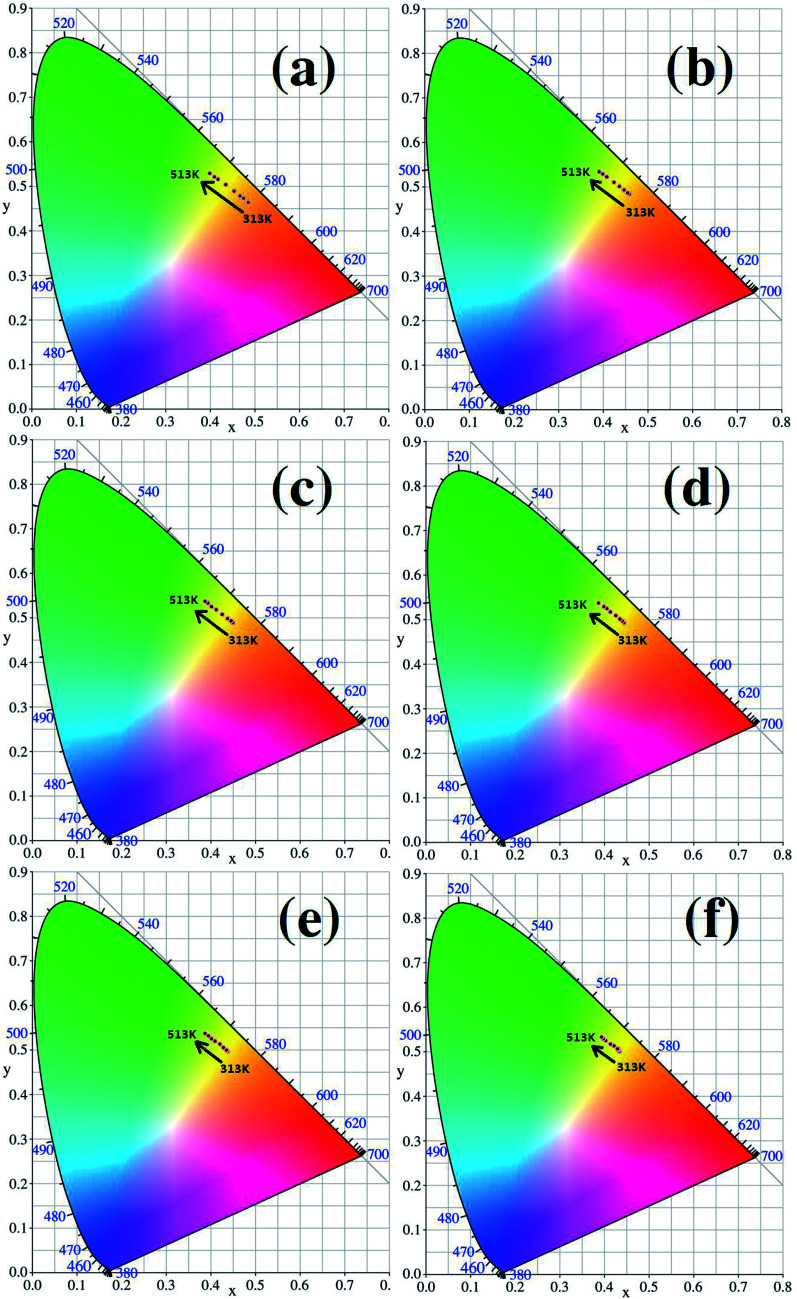
CIE coordinate diagrams for each sample (the particle sizes of the samples were (a) 200 nm, (b) 150 nm, (c) 100 nm, (d) 80 nm, (e) 50 nm and (f) 30 nm, respectively).

The CIE (Commission International del’ Eclairage) color coordinates of the Y_2_O_3_: 5% Tb^3+^, 0.5% Eu^3+^ phosphors with different particle sizes were calculated at 313–513 K, and the results are shown in Fig. 11(a–f). In Fig. 11, it can be see that as the temperature increases, the luminescent colors of the samples change from orange to green. So, the change in temperature is directly reflected by the change in the color of the luminescence. In addition, from Fig. 11, it can also be observed that the when particle size is larger, the range of the change in the color of the luminescence of the sample is greater, therefore the color of the luminescence changes more obviously with temperature.

## 4. Conclusions

In this paper, urea was used as a precipitation agent to synthesize Tb^3+^, Eu^3+^ co-doped Y_2_O_3_ nanophosphors by way of a homogeneous precipitation method. The size of the sample particles was controlled by changing the molar ratio of urea and rare earth ions. The particle size of the Y_2_O_3_: Tb^3+^, Eu^3+^ phosphor was observed to be around 200, 150, 100, 80, 50, and 30 nm when the molar ratio of urea and rare earth ions was 10, 20, 30, 60, 100 and 200, respectively. The optical temperature sensing properties of Y_2_O_3_: 5% Tb^3+^, 0.5% Eu^3+^ phosphors with different particle sizes were studied. The research shows that the FIR of Tb^3+^ to Eu^3+^ increased upon an increase in the temperature for all sized samples. Therefore, it was identified that FIR can be used to characterize temperature. Because of surface effects, the sensitivity was observed to increase upon a decrease in the particle size. However, due to size limitation effects, the relative sensitivity value decreased upon a decrease in the sample particle size. In addition, we also found that the luminescence color of the sample changed from orange to green when the temperature ranged from 313–513 K. So, the change in temperature can be directly reflected by the change in the luminescence color. The above results show that this work provides help for the study of the influence of size limitation and surface effects on the optical temperature sensing properties of nanomaterials.

## Conflicts of interest

There are no conflicts of interest to declare.

## Supplementary Material
